# Towards the Development of a Minigenome Assay for Species A Rotaviruses

**DOI:** 10.3390/v16091396

**Published:** 2024-08-31

**Authors:** Ola Diebold, Shu Zhou, Colin Peter Sharp, Blanka Tesla, Hou Wei Chook, Paul Digard, Eleanor R. Gaunt

**Affiliations:** Virology Division, Roslin Institute, University of Edinburgh, Easter Bush Campus, Midlothian EH25 9RG, UK

**Keywords:** rotavirus, minigenome, RNA-dependent RNA polymerase, reporter assay

## Abstract

RNA virus polymerases carry out multiple functions necessary for successful genome replication and transcription. A key tool for molecular studies of viral RNA-dependent RNA polymerases (RdRps) is a ‘minigenome’ or ‘minireplicon’ assay, in which viral RdRps are reconstituted in cells in the absence of full virus infection. Typically, plasmids expressing the viral polymerase protein(s) and other co-factors are co-transfected, along with a plasmid expressing an RNA encoding a fluorescent or luminescent reporter gene flanked by viral untranslated regions containing *cis*-acting elements required for viral RdRp recognition. This reconstitutes the viral transcription/replication machinery and allows the viral RdRp activity to be measured as a correlate of the reporter protein signal. Here, we report on the development of a ‘first-generation’ plasmid-based minigenome assay for species A rotavirus using a firefly luciferase reporter gene.

## 1. Introduction

Rotaviruses (RVs) are segmented, double-stranded RNA (dsRNA) viruses that cause acute gastroenteritis in infants, young children and livestock worldwide [1]. Currently, there are 11 distinct RV species (A–L, no E) with species A being the most predominant, accounting for over 90% of infections in humans and animals [2,3,4,5,6].

The RV virion is a non-enveloped triple-layered particle (TLP) containing 11 segments of dsRNA as its genome [7,8]. The core shell is formed by 60 asymmetric dimers of the viral protein 2 (VP2) and is surrounded by an intermediate layer of VP6 forming the transcriptionally active, non-infectious double-layered particle (DLP) [8,9]. The polymerase complex, composed of the RNA-dependent RNA polymerase (RdRp) (VP1) and the RNA-capping enzyme (VP3), is anchored at the five-fold axes through simultaneous interactions with multiple subdomains of VP2 [10,11]. The dsRNA segments are thought to be organised within the core in a way that each genome segment interacts with one specific polymerase complex [9,12].

The RdRp has a cage-like structure, with four tunnels leading to a catalytic core (residues 333–778) enclosed between the N-terminal (residues 1–332) and C-terminal domains (residues 779–1088) [13]. During transcription, the RdRp synthesises the capped, non-polyadenylated, positive-sense RNA ((+)RNA) transcripts from the minus strand of the genomic dsRNA, which are extruded out of the DLP into the cytoplasm [14]. The (+)RNA functions as mRNA for viral protein translation and as templates for the synthesis of new dsRNA genomes [15]. The cap-binding site of the N-terminal domain of VP1 splits the dsRNA genome through its interaction with the 5′ conserved m7GpppGGC residue of (+)RNA present in all the RV segments [16,17]. After a short part of the helix is unwound, the unpaired negative sense RNA ((−)RNA) traverses towards the active site of the RdRp and immediately pairs with complementary NTPs within the core that form a backbone of the nascent RNA [18]. The dsRNA genome is pushed along by the newly synthesised nascent RNA backbone until it reaches the C-terminal domain of VP1, where the coding strand reanneals with the template and reforms the dsRNA genome [13]. The presence of distinct exit tunnels ensures that the nascent RNA is released into the cytoplasm while the (−)RNA is reused in subsequent rounds of (+)RNA synthesis [13,19].

In RVs, highly conserved *cis*-acting elements that enhance (−)RNA synthesis were also shown to be present at the 5′-end of (+)RNA, which sometimes extended into the coding region [17,20,21,22,23]. Previous studies using in vitro replication systems showed that the complementary base pairing of 5′ and 3′ regions of each segment is predicted to facilitate RNA circularisation by forming panhandle structures where the 3′-GACC conserved terminal sequence extends as a single-stranded tail [17,24,25]. The RdRp specifically recognises the conserved consensus sequences at the 3′-end of (+)RNA to initiate (−)RNA synthesis during genome replication [16]. This interaction is catalytically inactive and requires the N-terminal domain of VP2, which leads to conformational changes in the priming loop within the catalytic core of VP1, stabilising the initiating nucleotide in the priming site of RdRp [26,27,28,29]. This correct alignment results in the formation of the first phosphodiester bond of the (−)RNA product [30]. Simultaneously, the priming loop retracts, allowing elongation of the dsRNA product out of the polymerase [13]. The ratio of VP1:VP2 required to achieve this replicase activity was shown to be 1:10, the same ratio that forms the vertices of the core [17,31]. Studies showed that assembly of VP2 into cores was required for RNA replication and encapsidation of VP1 and VP3, demonstrating its direct role in core assembly and the packaging of newly made dsRNA products [16,29].

The above understanding is derived from in vitro biochemistry experiments, which typically require laborious protein purification and which cannot easily interrogate host interactions or rapidly test mutant viral polypeptides. While reverse genetics systems for RVs now exist [32,33,34,35], they do not easily allow separate interrogation of viral transcription. For other Baltimore groups, the study of the RdRp function of plus-strand viruses is readily accessible due to minigenome assays developed in the 1990s [36], with the development of assays for the study of minus-strand virus RdRps following shortly thereafter [37,38,39]. Thus, the lack of a minigenome system for dsRNA viruses is a major roadblock to the study of the RdRps of dsRNA viruses.

In this study, we aimed to establish a plasmid-based minigenome assay to recapitulate viral transcription and replication, using a luciferase reporter construct flanked by viral UTRs. We found that the luciferase signal could be generated by expressing the subset of viral structural proteins required for DLP formation, if the ratios of the VP1:VP2 constructs were optimised. Mutations of the conserved residues in the catalytic core of the VP1 RdRp only modestly reduced the reporter activity but negated the virus rescue, suggesting that the reporter signal may also be amplified by viral proteins other than the VP1 RdRp. This new ‘first generation’ plasmid-based minigenome system has established a potential model for measuring the polymerase activity in vitro, with several avenues for how to improve this system now possible.

## 2. Materials and Methods

**Reporter segment construction.** The constructs were designed to encode the firefly luciferase gene in either a positive or negative orientation, flanked by 5′- and 3′-UTRs under a bacteriophage T7 RNA promoter (T7P), containing sequences for antigenomic hepatitis delta virus (HDV) ribozyme and T7 transcription terminator (T7T) sequences at the 3′-end. These constructs were synthesised by Invitrogen GeneArt on pMA (ampicillin resistance) vectors. The plasmids were amplified by transformation into chemically competent *E. coli* DH5α and purified using the QIAGEN^®^ Plasmid Midi Kit (QIAGEN, Manchester, UK) according to the manufacturer’s protocol. The inserts in each plasmid were verified by Sanger sequencing (GATC Biotech or Genewiz, Germany) using the primers listed in Table 1. The sequence results were analysed in SSE v1.4 software [40].

**Cell lines.** BSR-T7 cells, a derivative of baby hamster kidney fibroblasts (BHK-21 cells), constitutively expressing T7 RNA polymerase, were cultured in complete cell culture medium consisting of Glasgow’s Minimal Essential Medium (GMEM) (Gibco) supplemented with 1% tryptose phosphate broth (TPB) (Gibco), heat inactivated 10% foetal bovine serum (FBS) (Gibco) and 1% penicillin–streptomycin (Gibco). The cells were a kind gift from the laboratory of Prof. Massimo Palmarini (MRC-University of Glasgow Centre for Virus Research, UK). The cells were passaged twice weekly and maintained at 37 °C, 5% CO_2_. At every fifth passage, the G-418 selection drug (1 mg/mL) (Scientific Laboratory Supplies) was added to the cell media.

**Site-directed mutagenesis.** Site-directed mutagenesis was performed on the RF VP1 plasmid using the QuikChange II Site-Directed Mutagenesis Kit (Agilent Technologies, Cheadle, UK) according to the manufacturer’s instructions but using half-volume reactions. The thermal cycling parameters were as follows: 2 min denaturing at 95 °C, followed by 18 cycles of 30 s denaturing at 95 °C, 1 min primer annealing at 55 °C, 6 min elongation at 68 °C, with final 10 min elongation at 68 °C. The PCR products were digested using 1 µL of DpnI restriction enzyme to remove parental methylated DNA before transformation into competent *E. coli* cells. The products were visualised using gel electrophoresis. Successful mutagenesis was confirmed by Sanger sequencing.

**Virus rescue.** RV RF strain viruses and derivatives thereof were recovered using our previously described protocol [32]. In summary, BSR-T7 cells in 6-well plates were co-transfected with 11 plasmids corresponding to each RV genome segment (2.5 µg for plasmids encoding NSP2 and NSP5; 0.8 µg for the remaining plasmids) using 16 µL Lipofectamine 2000 (Invitrogen) per transfection. After 24 h incubation, MA104 cells (1 × 10^5^ cells/well) were added to the transfected BSR-T7 cells and co-cultured for 4 days in FBS-free Dulbecco’s Modified Eagle Medium (DMEM) (Sigma-Aldrich, Gillingham, UK) supplemented with 0.5 µg/mL porcine pancreatic trypsin type IX (Sigma-Aldrich). The co-cultured cells were then lysed three times by freeze/thaw and the lysates were incubated with trypsin at a final concentration of 10 µg/mL for 30 min to activate the virus. The lysates were then transferred to fresh MA104 cells in T25 flasks with 0.5 µg/mL porcine pancreatic trypsin type IX for up to 7 days and the viruses were harvested. Mock preparations with the mutated segment omitted were generated for use as negative controls throughout. All the rescue experiments were performed three times for each virus. The viruses were titred by plaque assays, and the presence of mutations in the VP1 gene segment was confirmed by Sanger sequencing (GATC Biotech or Genewiz, Germany).

**Plaque assay.** Plaque assays for RVs were performed using adapted methods [41,42]. Confluent monolayers of MA104 cells in 6-well plates were washed with FBS-free DMEM and infected with 800 μL of ten-fold serially diluted virus for 1 h at 37 °C 5% CO_2_. Following virus adsorption, 2 mL/well overlay medium was added (1:1 ratio of 2.4% Avicel (FMC Biopolymer, Philadelphia, USA) and FBS-free DMEM supplemented with 0.5 μg/mL porcine pancreatic trypsin type IX) and incubated for 4 days. The cells were then fixed for 1 h with 1 mL/well of 10% neutral buffered formalin (CellPath, Newtown, UK) and stained for 1 h with 0.1% Toluidine blue (Sigma-Aldrich) dissolved in H_2_O.

**Luciferase assay.** At 70–80% confluency, BSR-T7 cells in a 24-well plate were transfected with plasmid DNA using Lipofectamine 2000 reagent (Invitrogen, Loughborough, UK) according to the manufacturer’s protocol. Plasmid DNA and Lipofectamine reagent (1 µL of Lipofectamine for 1 µg of DNA) were separately diluted in 50 µL Opti-MEM. After a 5 min incubation at room temperature, the diluted mixes were combined and incubated for a further 25 min. During this time, the complete cell culture medium was changed to Opti-MEM (200 µL/well) and transfection mix was added to cells dropwise, which were then incubated for 48 h at 37 °C, 5% CO_2_. Following incubation, the supernatant was removed and the cells were lysed in 150 µL of Active Lysis Buffer (Promega, Chilworth, UK). The luciferase activity was analysed using Luciferase Assay Reagent (Promega) and the signal was read on a Cytation 3 plate reader (BioTek, Vermont, USA). A positive control plasmid expressing the Fluc gene driven by the cytomegalovirus (CMV) immediate early promoter, pVR1255, was used as a positive control throughout and is referred to as ‘+ve’.

**Statistical analysis.** GraphPad Prism v9 was used for all the statistical analyses. Data are presented as the mean and standard error of the mean from at least three independent experiments with technical duplicates unless otherwise stated. The *p* values were determined by ratio-paired *t*-test and were considered statistically significant at <0.05.

## 3. Results

**Figure 1:**
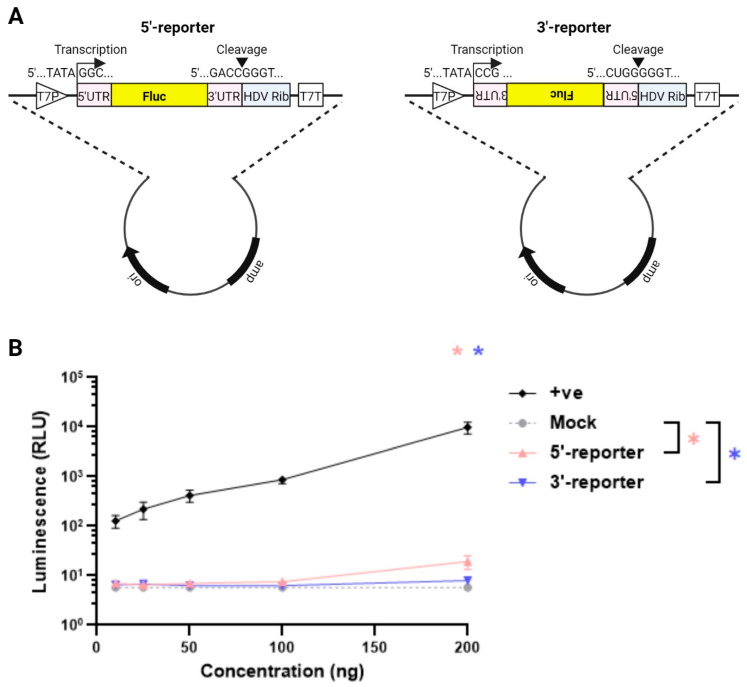
Minigenome reporter gene construct design. (**A**) Schematic of the reporter gene construct for the minigenome assay. Plasmids under a T7 promoter (T7P) encoding the Fluc gene in either the positive (5′-reporter) or negative sense (3′-reporter) flanked by the 5′- and 3′-UTRs. The plasmids included HDV ribozyme and T7 terminator sequences (T7T). Pink boxes, UTRs; yellow box, firefly luciferase ORF; blue box, HDV ribozyme sequence. (**B**) Dose-dependent titration of the 5′- and 3′-reporter plasmids. The pVR1255 plasmid expressing the Fluc gene was used as a positive control (denoted as ‘+ve’). The mock sample contained transfection reagent only. Data are the mean ± SEM from four independent experiments.

**Minigenome reporter construct design.** The highly conserved *cis*-acting signals in the 5′- and 3′-UTRs of all the RV segments are recognised by the viral RdRp for (+)RNA synthesis and dsRNA replication [16,17]. To set up a plasmid-based minigenome assay, synthetic DNA constructs were initially designed to express the firefly luciferase (Fluc) gene in either a positive or negative orientation flanked by the 5′- and 3′-UTRs from the RF strain NSP1 gene (Figure 1A; positive and negative sense reporters were named 5′-reporter and 3′-reporter respectively). NSP1 was selected by analogy with the influenza A virus minigenome assay, in which the open reading frame of NS1, also a broadly acting interferon pathway antagonist, is replaced by a reporter gene [43]. The viral UTRs were flanked by T7P and HDV ribozyme sequences, as used successfully in the development of the RV reverse genetics system [33]. Thus, transcription of the resulting vector would generate full length viral (+) single-stranded RNA transcripts containing native viral 5′ and 3′ termini [44]. If these RNAs were transcribed and/or replicated by the viral RdRp, the luciferase levels in the transfected cells would be expected to increase.

To determine the quantity of the reporter plasmids required that could be transfected while generating only minimal background signal, increasing amounts were transfected into BSR-T7 cells (Figure 1B). The positive control (‘+ve’) luciferase-expressing plasmid produced a strong luciferase signal that increased with the plasmid dose. In contrast, the RV reporter genes gave very low levels of signal, not significantly above the background of no luciferase gene at doses of 100 ng and lower (Figure 1B). Both reporters gave levels of signal that were above background at 200 ng, but this was still around 30 RLU or lower, so this was chosen as the amount of reporter construct to take forward for further assay development.

**Figure 2:**
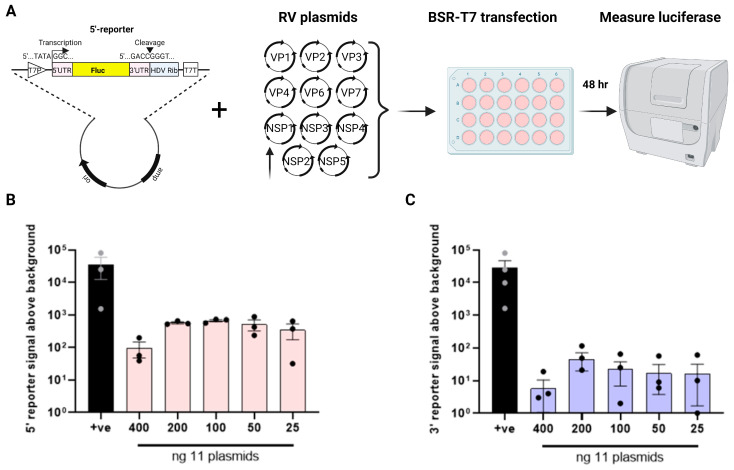
Reporter expression by rotavirus polymerase. (**A**) Schematic of the proposed minigenome assay. RV plasmids encoding each bovine RF stain gene were co-transfected with T7 reporter plasmids expressing the Fluc gene in either the positive (5′-reporter) or negative sense (3′-reporter). Luciferase activity was measured after 48 h post transfection. Dose-dependent titration of 11 RV plasmids with 200 ng of the 5′-reporter in (**B**) and with 200 ng of the 3′-reporter in (**C**), with the RLU values for the reporter-only signal (‘background’) subtracted. As in reverse genetics, the amount of plasmids expressing NSP2 and NSP5 genes was increased to scale.

**Reporter expression by RV polymerase.** During RV reverse genetics, 11 plasmids (1 per genome segment) are co-transfected into BSR-T7 cells for successful virus rescue, meaning that viral polymerase is functional and is able to copy both transcript polarities, thereby generating the complete viral genome. We therefore predicted that co-transfection of either of our luciferase reporters with the full complement of reverse genetics plasmids would result in an amplification of the luciferase signal above the background. To test this, a dose-dependent titration was performed for all 11 plasmids in the presence of 200 ng of the 5′- and 3′-reporters to determine the minimal amount of RV plasmids needed to generate the strongest luciferase signal (Figure 2A; as in reverse genetics [32,34], NSP2 and NSP5 plasmids were used in 3.125X amounts relative to the other nine plasmids). The pVR1255-positive control plasmid produced a strong luciferase signal (Figure 2B,C). Throughout, both 5′- and 3′-reporter plasmids expressed alone gave similar levels of background to those seen in Figure 1B, and so the cognate background reporter signals were subtracted from all the readings from samples transfected with RV plasmids. The 5′-reporter yielded a 2–3 log_10_ increase in luminescence above the background between 25 and 200 ng of the 11 RG plasmids, with apparent saturation at 200 ng and a decrease at 400 ng (Figure 2B). The same trend was observed for the 3′-reporter, but the overall luminescence signals were around one log_10_ lower than for the 5′-reporter. The 5′-reporter was therefore considered to be more suitable than the 3′-reporter for the minigenome assay, with 100–200 ng (312.5–625 ng for NSP2 and NSP5) of each reverse genetics plasmid being the optimal amount.

**Figure 3:**
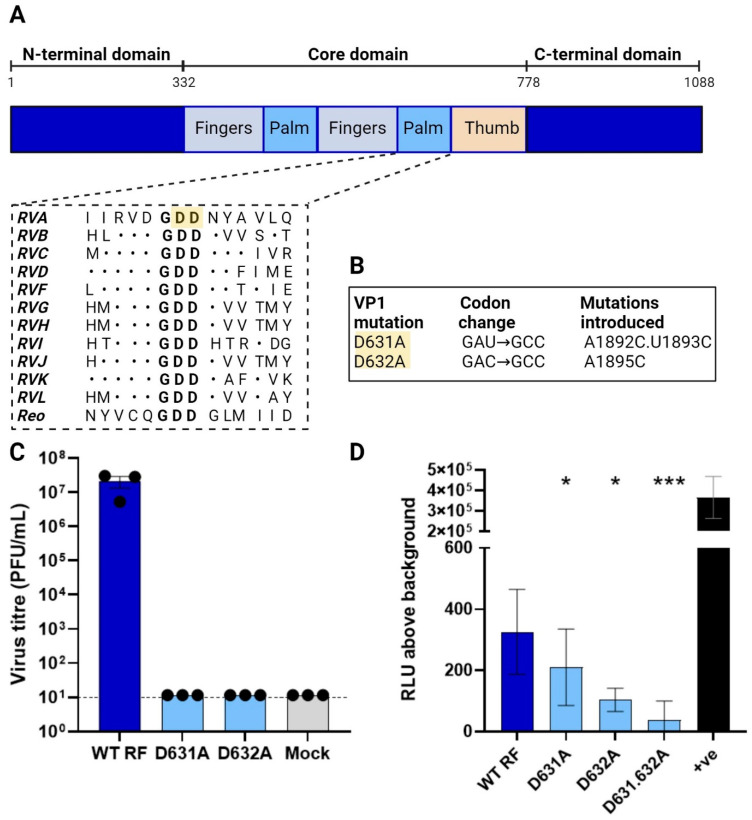
Generation of inactive polymerase. (**A**) VP1 shown as a linear schematic and coloured according to the domain organisation, with amino acid numbers labelled above. Adapted from [27], with alignments for RVH-RVL updated/added based on their reference sequences (KT962027.1, NC_026825.2, NC_055268.1, OQ934016.1, OM101015.1). The N-terminal and C-terminal domains (deep blue) flank the core domain containing the fingers (pale blue), palm (mid blue) and thumb (orange). Sequence-based alignment of RdRps across RV species and the related reovirus showing the conserved catalytic ‘GDD’ site. Dots indicate amino acid conservation. Highlighted in yellow are the two conserved aspartic acid residues targeted for mutagenesis. (**B**) Mutagenesis strategy for evolutionarily conserved aspartic acid residues in the VP1 catalytic domain. (**C**) Viral titres of WT RF and of VP1 mutants. (**D**) Minigenome assay for VP1 mutants. In all cases, all 11 RG plasmids were transfected (with 3.125×X amounts of NSP2 and NSP5 plasmids) along with 200 ng 5′-reporter. pVR1255 plasmid expressing Fluc gene was used as a positive control (denoted as ‘+ve’). The RLU values for the reporter-only signal (‘background’) were subtracted. * *p* < 0.05; *** *p* < 0.001.

**Generation of an inactive RdRp.** The increase in the luciferase signal in the presence of all the RV polypeptides was suggestive but not conclusive evidence of viral polymerase activity. Therefore, for further assay validation, we sought to generate a viral RdRp VP1 mutant lacking polymerase activity. The RV RdRp resembles a ‘right-handed’ architecture made up of the N-terminal domain, the core and the C-terminal domain, where the core is further split into the ‘palm, finger and thumb’ subdomains [19,27]. Ogden et al. (2012) showed that the conserved aspartate residues within the ‘GDD’ motif in the palm subdomain (Figure 3A) were critical for RNA synthesis [26]. These conserved aspartate residues, D631 and D632, were mutated to alanine by site-directed mutagenesis, creating the mutants D631A and D632A, respectively (Figure 3B). To test for successful inhibition of viral RdRp activity, rescues of the VP1 mutants were attempted using reverse genetics, and as expected, no virus was recovered in the presence of the mutated polymerase (Figure 3C). This confirmed that the mutations rendered the virus replication incompetent. The same VP1 mutants were therefore tested in the minigenome assay, with 11 RV gene segments (100 ng each except 312.5 ng for NSP2 and NSP5), co-transfected into BSR-T7 with 200 ng of the 5′-reporter. As before, a pVR1255-positive control produced a strong luciferase signal (Figure 3D). Unexpectedly, however, only a modest decrease in signal was observed for the D631A mutant and only the D632A mutant yielded a significant reduction. Thus, while the entire GDD motif appears to be essential for viral rescue, single amino acid mutations were tolerated in the minigenome assay. Combining D631A and D632A mutations emphasised the reduction in luciferase signal, but this was still above the background (Figure 3D). The VP1 mutants possibly retained some polymerase activity, or some of the luciferase signal was generated by other viral proteins.

**Figure 4:**
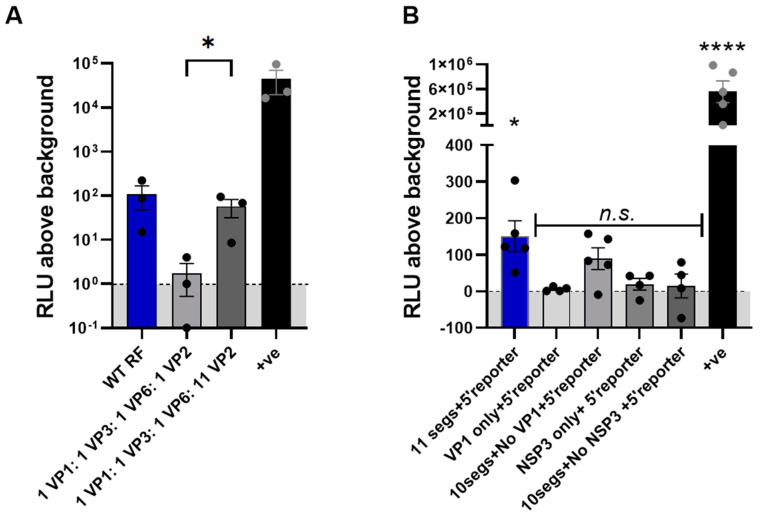
Measuring RdRp activity. Luciferase activity following co-transfection of RV plasmids with 200 ng of 5′-reporter. WT RF denotes co-transfection of all 11 RG plasmids (with 3.125× amounts of NSP2 and NSP5 plasmids) with the reporter. The pVR1255 plasmid expressing the Fluc gene was used as a positive control (denoted as ‘+ve’). The RLU values for the reporter-only signal (‘background’) were subtracted. (**A**) To test whether the number of RG plasmids expressed could be reduced, 4 plasmids corresponding to VP1, VP2, VP3 and VP6 were co-transfected with the 5′-reporter (‘4 plasmids + 5′ rep). To test whether the 4-plasmid system could be improved upon, the amount of the VP2 plasmid was increased 11-fold (‘VP1:VP2 ratio’). (**B**) To test the dependency of the system on VP1 and NSP3, polymerase assays were undertaken either by co-transfecting reporter with VP1 or NSP3 alone, or with ten segments minus either VP1 or NSP3. * *p* < 0.05; **** *p* < 0.0001; *n.s*, not significant.

**Exploring the minimal requirements for the RV minigenome assay.** Although 11 plasmids are required for viral rescue, we considered the possibility that not all 11 segments may be needed for the minigenome assay. During RV replication, upon cell entry, the loss of the outer protein layer of VP4 and VP7 triggers a conformational switch that induces polymerase activity in the now double-layered virus particle (DLP) [45]. The DLP contains a core comprising VP1, VP2 and VP3, surrounded by a shell of VP6. We therefore considered the possibility that reconstitution of the DLP alone may be sufficient for polymerase activity and whether transfection of constructs delivering only these four proteins with the 5′-reporter was sufficient to yield a luciferase signal. However, transfecting equimolar amounts of the VP1-3 and VP6 plasmids resulted in a dramatic loss of signal relative to the 11-plasmid system that was barely above the background (Figure 4A). Patton et al. (1997) showed that a molar ratio of 1 VP1 to 11 VP2, similar to that found in virion cores, produced the highest level of dsRNA synthesis in the cell-free system [46]. Therefore, we also tested whether adjusting the VP1:VP2 ratio to 1:11 would improve the signal. Indeed, when the amount of VP2 plasmid was increased 11-fold, the signal increased significantly and was only slightly lower than that of the 11-plasmid system.

We next sought to determine whether VP1 RdRp alone could support amplification of the reporter signal, and whether NSP3 would serve to enhance the translation. To test this, each of these constructs was expressed either alone with the luciferase reporter or reporter was expressed with constructs representing the remaining ten segments in the absence of VP1 or NSP3. However, in none of these cases was the signal significantly above the background (Figure 4B).

## 4. Discussion

Minigenome assays have been used to study the in-cell polymerase activity of a range of RNA viruses with single-stranded genomes, including influenza A virus [46], respiratory syncytial virus [47] and poliovirus [48]. As far as we are aware, in-cell reconstitution of viral polymerase activity has not been achieved previously for a virus with a double-stranded RNA genome, although polymerase activity has been assayed in a cell-free system for bluetongue virus [49]. Minigenome assays have yielded significant breakthroughs in the understanding of viral polymerase functions and domains, and this development represents an opportunity for such research questions to be applied to RVs.

Through this work, we unexpectedly found that mutating the highly conserved catalytic GDD motif of RV VP1 only modestly reduced the luciferase signal (Figure 3D). This may suggest that a significant part of the luciferase signal generated in the polymerase assay is attributable to the activity of viral protein(s) other than VP1. When either VP1 or NSP3 were excluded from the system but the remaining ten viral proteins were co-expressed with the reporter, the luciferase signal was not significantly above the background (Figure 4B), although some signal was observed in the ‘no VP1’ transfection. As exclusion of NSP3, or expression of NSP3 or VP1 alone, did not increase the reporter signal above the background, this suggests that if the reporter signal is indeed generated by viral proteins other than the VP1 polymerase, the way this arises is complicated by interactions between multiple viral proteins. The minor reduction in the luciferase signal brought about by the D631A and D632A mutations in VP1 is likely insufficient to explain why the same mutations abrogated virus production. Possibly, this domain of VP1 has multiple functions required to complete a virus lifecycle beyond its well-characterised role in dsRNA replication, such as genome packaging [19,26].

We have demonstrated that the reporter signal is generated when only components of the DLP are expressed, with no absolute requirement for non-structural proteins. NSP2 and NSP5 are together necessary and sufficient for the formation of viroplasms (or viroplasm-like structures), which form a sequestered environment for the accumulation of viral proteins and DLPs [45]. NSP3 replaces poly-A-binding protein in ribosomal complexes to bind viral non-polyadenylated transcripts, thereby enhancing their translation (and also reducing the translation of cellular polyadenylated transcripts) [50]; nevertheless, in its absence, viral polymerase activity was apparent (Figure 4A), demonstrating that translation of viral proteins occurs readily in NSP3’s absence. To improve the dynamic range of this assay, co-expression of this subset of non-structural proteins could be explored. Co-expression of capping enzymes such as that of African swine fever virus, shown to be efficient in RV reverse genetics [51], may also augment the efficiency of this minigenome assay. Alternatively, it may be possible to reduce the number of plasmids required; we have shown that a signal can be generated when expressing only VP1, 2, 3 and 6, but it may be possible to reduce this further by removing VP 3 and/or 6; expression of VP1 alone did not yield a reporter signal significantly above the background (Figure 4B).

Further improvements to this system might include the co-expression of multiple viral gene segments from the same plasmid. This would reduce the number of plasmids being co-transfected and so theoretically increase the number of cells receiving the full complement of viral proteins required for viral polymerase activity to occur. There may be a trade-off due to the possibility of larger plasmids transfecting with poorer efficiency, but we have found that analogous multi-segment plasmids improve the minigenome assay efficiency in the influenza A virus system (unpublished data).

The polymerase assay system reported here was established using reverse genetics plasmids and so all viral gene segments were under a T7 promoter, necessitating the use of BSR-T7 cells. It is likely that higher transfection efficiencies would be achieved in HEK293T cells (infectable with RV in our hands), which are used for other minigenome assays, including that of influenza A virus. Testing this would require cloning of the constructs used into a different backbone so that viral genes are expressed under a mammalian promoter such as CMV. Alternatively, the T7 polymerase would need to be expressed in HEK293T cells.

Here, we have established a ‘first generation’ minigenome assay for the RF strain, which is a widely used and well-characterised lab strain of RV. The generalisability of this approach to other RV strains should be tested in the future using the analogous approach of generating a reporter construct encoded by flanking NSP1 UTRs of the cognate strain, analogous to strategies for maximising influenza A virus gene expression [52]. It is possible that the use of UTRs from other viral segments would yield a higher translational efficiency, which was not examined here. Reporter constructs comprising the UTRs from heterologous RV strains should also be explored as this would negate the requirement for strain-specific reporter constructs.

## Figures and Tables

**Table 1:** Sequences of primers used in this study.

Target gene	Sequence (5′ to 3′)	Use
Fluc in pMA plasmid	TAATACGACTCACTATAGGG TCGTCCACTCGGATGGCTA	Sequence 5′- and 3′-plasmids containing Fluc gene
VP1 plasmid	GGAAGGAGAGATGTACCAGGA	Sequence mutations of GDD motif in VP1 plasmid

## Data Availability

All data presented in this manuscript are available at the discretion of the corresponding authors.
